# Latent heterogeneity of muscle‐invasive bladder cancer in patient characteristics and survival: A population‐based nation‐wide study in the Bladder Cancer Data Base Sweden (BladderBaSe)

**DOI:** 10.1002/cam4.5981

**Published:** 2023-04-25

**Authors:** Christel Häggström, Mark Rowley, Fredrik Liedberg, Anthony C. C. Coolen, Lars Holmberg

**Affiliations:** ^1^ Department of Surgical Sciences Uppsala University Uppsala Sweden; ^2^ Northern Registry Centre, Department of Public Health and Clinical Medicine Umeå University, Umeå University Umeå Sweden; ^3^ Translational Oncology & Urology Research (TOUR) School of Cancer and Pharmaceutical Sciences, King's College London UK; ^4^ Saddle Point Science York UK; ^5^ Saddle Point Science Europe Nijmegen The Netherlands; ^6^ Department of Urology Skåne University Hospital Malmö Sweden; ^7^ Institution of Translational Medicine Lund University Malmö Sweden; ^8^ Department of Biophysics, Faculty of Science, Donders Institute Radboud University Nijmegen The Netherlands

**Keywords:** bladder cancer, competing risks, latent class, survival analysis

## Abstract

**Background:**

Patients with muscle‐invasive bladder cancer (MIBC) constitute a heterogenous group in terms of patient and tumour characteristics (‘case‐mix’) and prognosis. The aim of the current study was to investigate whether differences in survival could be used to separate MIBC patients into separate classes using a recently developed latent class regression method for survival analysis with competing risks.

**Methods:**

We selected all participants diagnosed with MIBC in the Bladder Cancer Data Base Sweden (BladderBase) and analysed inter‐patient heterogeneity in risk of death from bladder cancer and other causes.

**Results:**

Using data from 9653 MIBC patients, we detected heterogeneity with six distinct latent classes in the studied population. The largest, and most frail class included 50% of the study population and was characterised by a somewhat larger proportion of women, higher age at diagnosis, more advanced disease and lower probability of curative treatment. Despite this, patients in this class treated with curative intent by radical cystectomy or radiotherapy had a lower association to risk of death. The second largest class included 23% and was substantially less frail as compared to the largest class. The third and fourth class included each around 9%–10%, whereas the fifth and sixth class included each 3%–4% of the population.

**Conclusions:**

Results from the current study are compatible with previous research and the method can be used to adjust comparisons in prognosis between MIBC populations for influential differences in the distribution of sub‐classes.

## 1 INTRODUCTION

Despite patients with muscle‐invasive bladder cancer (MIBC) constituting a very heterogenous group in terms of tumour characteristics, age at diagnosis, gender, comorbidities and socioeconomic background factors,^1^, ^2^, ^3^ very few studies have investigated whether these differences can be used to separate MIBC patients into distinct classes with possible differences in prognosis.^4^, ^5^

Characterisation of such classes could help individualise treatment to avoid under‐as well as over‐treatment if they are associated with response to treatment. Characterising the clinical implications of classes can be a tool for better understanding of how MIBC cohorts develop over time: for example one class with longer survival will come to dominate the cohort over time after exhaustion of another class with bad prognosis. Furthermore, separation into classes can increase our understanding of the differences in case‐mix of MIBC patients that influence comparisons in observational studies of performance or survival between countries, regions, or hospitals.

A tool for assessing heterogeneity is to include the concept of frailty, which could further enhance prognostication and identify more vulnerable patients. For prognosis of MIBC, different clinical scores assessing frailty based on known risk factors have already been proposed.^6^, ^7^ In the current study, we used a recently developed method to investigate frailty based on factors that are not present in the data as covariates, that is unmeasured and hitherto unknown covariates associated to survival, in addition to account for risk of competing causes of death, which has to our knowledge never been investigated previously in MIBC patients.

The primary research question of the current study is to determine whether we could identify latent classes of MIBC patients by using data from a nation‐wide bladder cancer database in Sweden and applying a novel statistical approach to latent class proportional hazards models and if so, compute their class‐specific characteristics, frailty and associations to risk of death from bladder cancer and from other causes. A secondary aim was to investigate if the distribution of classes were differently geographically distributed.

## 2 METHODS

### 2.1 Study population

We selected all participants diagnosed with MIBC (defined as T‐stage ≥2) in the Bladder Cancer Data Base Sweden (BladderBaSe).^8^ The BladderBaSe started in 2014 by merge of the Swedish National Registry for Urinary Bladder Cancer (SNRUBC) to several national health care and sociodemographic registers. Details of the multi‐register linkage and variables included can be found elsewhere.^8^ In short, we merged clinical data of all individuals with primary bladder cancer in the SNRUBC with data on socioeconomic factors, comorbidity, other clinical and cancer diagnoses, and cause and date of death. The project has been authorised by the Research Ethics Board at Uppsala University, Sweden (EPN Dnr 2015/277).

### 2.2 Definition of covariates

For this investigation we retrieved data from the SNRUBC on T, M and N stage and grade at diagnosis, type of treatment (no curative treatment, or curative treatment by radical cystectomy or radiotherapy), diagnosing hospital size (university, region or district hospital) and healthcare region (six different in Sweden). From the National Patient Register, we used data from hospital admissions up to 10 years prior to the date of bladder cancer diagnosis. These data on discharge diagnoses were used to compute the Charlson Comorbidity Index (CCI), and the CCI were categorised into four groups: no comorbidities, mild, moderate, or severe comorbidity (0, 1, 2 and ≥3 comorbidities^9^, ^10^). From the Longitudinal Integration Database for Health Insurance and Labor Market Studies, we retrieved data on educational level and categorised this into three groups: ≤9 years, 10–12 years and ≥ 13 years, which corresponded to low, intermediate and high education level.^11^ In all analyses type of treatment, gender (male or female), diagnosis age (continuous), calendar year at diagnosis (continuous), education level (in categories described above), CCI (in categories described above), hospital size, T stage at diagnosis (T2, T3 or T4), M stage at diagnosis (no distant metastases [M0], distant metastases [M1], or not measured [MX]), N stage at diagnosis (no regional lymph nodes with cancer [N0], cancer in at least one of the regional lymph nodes [N+] or not measured [NX]) and grade at diagnosis (G1, G2 or G3, according to World Health Organization 1973 classification from 1997 to 2003 and according to World Health Organization 1999 from 2004 and onwards) were used as covariates.

### 2.3 Definition of follow‐up and endpoints

Start date of the study was defined as date of diagnosis of MIBC, and last date of the study was date of death, or December 31, 2014, whichever occured first. Timescale for follow up were years from diagnosis. Date and cause of death were acquired from the Cause of Death Register. Death from bladder cancer were defined as code C67 (International Classification of Diseases, 10th revision). Bladder cancer death and death from other causes were used as endpoints in the analysis.

### 2.4 Statistical analysis

To investigate inter‐patient heterogeneity taking competing risks into account, we used latent class proportional hazards models. Detailed information of the latent class proportional hazards models can be found elsewhere.^12^ In short, latent class proportional hazards analysis was performed in two steps; the first step was to perform regression for models having a range of latent classes with different degrees of complexity. We used Akaike information criteria as the second step to select the model best supported by the evidence in the studied survival data, having the optimal number of sub classes and complexity. The model used does not assume homogenous associations between covariates and endpoints, accounting simultaneously for the risks of both endpoints, in comparison to models in conventional survival analysis. The model builds on the assumption that the study population may be composed of several latent classes, and that differences in risks of the endpoints between classes are caused by latent heterogeneity, that is heterogeneity not captured by the studied covariates. The latent heterogeneity between the classes can be quantified by relative frailty measures, that is variability in base hazard rates and associations. For each latent class, there is an assumption of proportional hazards for the associations with covariates to each endpoint in the study. Hence the assumption of proportional hazard is not required to be valid for the full study population. The method will result in a model similar to the Cox proportional hazards model if no latent classes are found in the population analysed.

We assessed differences in class membership, frailty and associations, based on the estimates of the latent class proportional hazards analysis. Frailty was defined as the heterogeneity resulting from risk factors that were not explicitly present in the survival data as covariates, that is unmeasured and hitherto unknown covariates associated with the risk of any of the endpoints. Associations with covariates and latent class membership on risk of all endpoints were investigated by calculations of hazard ratios (HR) from the latent class model for competing risk analysis. Relative frailties between classes detected by the model were assessed by HR for latent class membership. The analysis was carried out using the software package SaddlePoint‐Mosaics version 1.1.0 (https://www.saddlepointscience.com/) which implements the method as presented previously in the literature.^12^ The estimated latent classes were tabulated versus health care regions to investigate differences between the six Swedish health care regions (North, Mid, Stockholm/Gotland [Sthlm], West, Southeast and South).

## 3 RESULTS

The study population include 9653 patients, 71% men and 29% women diagnosed with MIBC, (Table 1 **)**. Mean age at start of study was 75 years (SD = 11), and during follow‐up, 5235 (54%) patients died of bladder cancer and 2235 (23%) died of other causes. Mean follow‐up time in the cohort were 2.6 years (SD = 3.5), and survival time (excluding patients alive at end of study period) were median 0.8 years (IQR = 0.4–1.8).

**TABLE 1:** Baseline characteristics of covariates included in the analysis of full study population (9653 patients with muscle‐invasive bladder cancer), and six latent classes as a result from the competing risk latent class analysis.

	Full study population (*N* = 9653, 100%)	Class 1 (*N* = 341, 3.5%)	Class 2 (*N* = 384, 4.0%)	Class 3 (*N* = 954, 9.9%)	Class 4 (*N* = 920, 9.5%)	Class 5 (*N* = 4795, 49.7%)	Class 6 (*N* = 2259, 23.4%)
Sex, *n* (%)							
Men	6809 (71)	215 (63)	241 (63)	676 (71)	667 (73)	3295 (69)	1715 (76)
Women	2844 (29)	126 (37)	143 (37)	278 (29)	253 (28)	1500 (31)	544 (24)
Curative treatment, *n* (%)							
No curative treatment	5436 (56)	243 (71)	253 (66)	630 (66)	556 (60)	2808 (59)	946 (42)
Radical cystectomy	3540 (37)	55 (16)	110 (29)	213 (22)	330 (36)	1623 (34)	1209 (54)
Radiotherapy	677 (7)	43 (13)	21 (5)	111 (12)	34 (4)	364 (8)	104 (5)
Age at diagnosis, *n* (%)							
Below 75 years	4378 (45)	158 (46)	129 (34)	353 (37)	379 (41)	2054 (43)	1305 (58)
75 years and above	5275 (55)	183 (54)	255 (66)	601 (63)	541 (59)	2741 (57)	954 (42)
Calendar year of diagnosis, *n* (%)							
1997–2000	2065 (21)	83 (24)	100 (26)	282 (30)	210 (22)	1085 (23)	314 (14)
2001–2005	2706 (28)	154 (45)	103 (27)	263 (28)	307 (33)	1330 (28)	519 (24)
2006–2009	2149 (22)	46 (13)	130 (34)	253 (27)	240 (26)	1076 (22)	404 (18)
2010–2014	2733 (28)	58 (17)	51 (13)	156 (16)	172 (19)	1304 (27)	992 (44)
Educational level^a^, *n* (%)							
Low^b^	5366 (56)	228 (67)	236 (61)	554 (58)	608 (66)	2755 (57)	985 (44)
Intermediate	3028 (31)	78 (23)	93 (24)	272 (29)	228 (25)	1472 (31)	885 (39)
High	1259 (13)	35 (10)	55 (14)	128 (13)	84 (9)	568 (12)	389 (17)
Charlson Comorbity Index, *n* (%)							
No comorbidity (0)	5450 (56)	162 (48)	166 (43)	481 (50)	453 (49)	2676 (56)	1512 (67)
Mild comorbidity (1)	1769 (18)	84 (25)	118 (31)	131 (14)	210 (23)	920 (19)	306 (14)
Moderate comorbidity (2)	1284 (13)	35 (10)	33 (9)	206 (22)	163 (18)	597 (12)	250 (11)
Severe comorbidity (3 or more)	115 (12)	60 (18)	67 (17)	136 (14)	94 (10)	602 (13)	191 (8)
T stage, *n* (%)							
T2	6444 (67)	161 (47)	190 (49)	652 (68)	591 (64)	3007 (63)	1843 (82)
T3	1951 (20)	111 (33)	119 (31)	181 (19)	234 (25)	1016 (21)	290 (13)
T4	1258 (13)	69 (20)	75 (20)	121 (13)	95 (10)	772 (16)	126 (6)
Grade, *n* (%)							
G2^c^	1266 (13)	39 (11)	59 (15)	137 (14)	161 (18)	559 (12)	311 (14)
G3	7848 (81)	277 (81)	299 (78)	761 (80)	715 (78)	3954 (82)	1842 (82)
GX/missing	539 (6)	25 (7)	26 (7)	56 (6)	44 (5)	282 (6)	106 (5)
M stage, *n* (%)							
M stage 0	4515 (47)	123 (36)	171 (45)	421 (44)	390 (42)	2117 (44)	1293 (57)
M stage 1	1006 (10)	53 (16)	25 (7)	76 (8)	101 (11)	622 (13)	129 (6)
M stage X^d^	4132 (43)	165 (48)	188 (49)	457 (48)	429 (47)	2056 (43)	837 (37)
N stage, *n* (%)							
N stage 0	3837 (40)	113 (33)	139 (36)	347 (36)	318 (35)	1751 (37)	1169 (52)
N stage +^e^	1149 (12)	66 (19)	23 (6)	87 (9)	121 (13)	677 (14)	175 (8)
N stage X^f^	4667 (48)	162 (48)	222 (58)	520 (55)	481 (52)	2367 (49)	915 (41)
Hospital size, *n* (%)							
University hospital	3256 (34)	109 (32)	124 (32)	273 (29)	284 (31)	1584 (33)	882 (39)
Region hospital	4443 (46)	145 (43)	174 (45)	410 (43)	415 (45)	2251 (47)	1048 (46)
District hospital^g^	1954 (20)	87 (26)	86 (22)	271 (29)	221 (24)	960 (20)	329 (15)

^a^ Educational level categorised as low (≤9 years of school), intermediate (10–12 years), and high (≥13 years), corresponding to mandatory school, high school, and college or university.

^b^ Educational level missing 514 men, these men were included in the group with low educational level.

^c^ Eighty patients categorised as G1 were added to G2.

^d^ Includes 87 patients with missing M stage.

^e^ Includes patients with N stage 1 to 3.

^f^ Includes 53 patients with missing N stage.

^g^ Includes 17 patients at hospitals not categorised.

Information on baseline characteristics and endpoints used in the calculations of latent class proportional hazards models and baseline characteristics included in the model computation are shown in Table 1. The analysis resulted in a model with six different latent classes as being best supported by the survival data in this study. We determined the characteristics, frailty and the associations to risks of the endpoints of the study participants in relation to the most probable latent class they were assigned to.

The largest class included 4795 individuals (49.7%) of the study population (Class 5) (Table 1). The second largest class included 2259 individuals (23.4%) (Class 6). The third and fourth largest class included 954 individuals (9.9%) (Class 3) and 930 individuals (9.5%) (Class 4). The smallest classes included 384 individuals (4.0%) (Class 2) and 341 individuals (3.5%) (Class 1).

Class 5 had a somewhat larger proportion of women, patients older than 75 years at diagnosis, more advanced bladder cancer tumour (by T, M and N stages and grade at diagnosis), and individuals not receiving curative treatment (Table 1). Thus, Class 5 included only 9.6% of patients that were alive at end of study period, and 76.8% of all patients that died of bladder cancer, albeit 25.3% died of other causes during the follow‐up (Table 2). Class 5 had a median survival of 0.5 years (IQR: 0.3–1.0 years), excluding patients alive at end of follow‐up. In Class 5, we found that radical cystectomy and radiotherapy were both associated with a decreased risk of both death from bladder cancer and other causes, as compared to no treatment (Table S1, for *p* < 0.001). Furthermore, T3 and T4 (as compared to T2), M1 (as compared to M0) and N+ (as compared to N0) were all associated with increased risk of death from bladder cancer, while NX (as compared to N0) were associated to risk of death from other causes (Table S1, for *p* < 0.001). As Class 5 was both the largest and the most frail class, we used that class as a reference group for calculations of frailty of all other classes for both endpoints studied.

**TABLE 2:** Status at end of study, survival times, frailty factors, relative frailty factors and HRs due to class membership (due to frailty) in the six latent classes, as compared to Class 5 (the largest class and also most frail) for both endpoints.

	Class 1 (*N* = 341, 3.5%)	Class 2 (*N* = 384, 4.0%)	Class 3 (*N* = 954, 9.9%)	Class 4 (*N* = 920, 9.5%)	Class 5 (*N* = 4795, 49.7%)	Class 6 (*N* = 2259, 23.4%)
Status at end of study (%)						
Alive	1.0	3.1	11.5	10.5	9.6	64.2
BC death	0.8	1.4	5.4	6.0	76.8	9.6
Other death	12.3	10.8	18.8	16.9	25.3	15.8
Survival time (years)^a^						
Median (IQR)	0.8 (0.2–2.1)	2.3 (1.2–5.2)	2.2 (1.2–4.5)	1.6 (0.9–3.9)	0.5 (0.3–1.0)	1.6 (0.9–3.5)
Frailty factor						
BC death	−2.20 (−1.80, −2.61)	−3.06 (−2.78, ‐3.34)	−3.17 (−2.90, −3.45)	−2.98 (−2.70, −3.26)	−0.35 (−0.20, −0.50)	−2.25 (−1.99, −2.50)
Other death	−0.68 (1.26, −2.61)	−2.02 (−1.39, −2.66)	−1.65 (−1.35, ‐1.95)	−2.31 (−1.99, ‐2.63)	−0.47 (−0.10, ‐0.84)	−2.08 (−1.73, −2.43)
Relative frailty factors^b^						
BC death	−1.85	−2.71	−2.82	−2.63	NA	−1.90
Other death	−0.21	−1.55	−1.18	−1.84	NA	−1.61
HR due to class membership^c^						
BC death	0.16	0.07	0.06	0.07	1, ref	0.15
Other death	0.81	0.21	0.31	0.16	1, ref	0.20

^a^ Excluding study participants alive at end of study.

^b^ Relative contribution due to class membership as compared to the largest class (Class 5), defined as relative frailty factor = abs[frailty (Class 5)]‐abs[frailty (Class X)].

^c^ Association due to class membership: HR = exp(relative frailty factor).

Class 6, the second largest class, had a higher proportion of men, below 75 years at diagnosis and with very few comorbidities (Table 1). Furthermore, this class had a higher proportion with higher education level, diagnosed at university hospitals, less advanced bladder cancer tumour (with respect to T, N and M stage, and grade) and were treated with radical cystectomy. Class 6 included 64.2% of the patients that were alive at end of study period, 9.6% of patients that died of bladder cancer and 15.8% of the that died of other causes (Table 2). Within Class 6, median survival time were 1.6 years (IQR: 0.9–3.5 years), and severe comorbidity (as compared to no comorbidities), T3 and T4 (as compared to T2) were associated to increased risk of bladder cancer death, furthermore, higher age at diagnosis and M1 (as compared to M0) were associated with risk of death from other causes. (Table S1, for *p* < 0.001). Thus, Class 6 were less frail (as compared to Class 5) with HRs at 0.2 and 0.15 for the endpoints, which implies that patients in Class 6 had a hazard rate of the endpoints of one fifth and almost one seventh as compared to Class 5.

Class 3 and Class 4, the third and fourth largest classes, were substantially smaller and more difficult to characterise (Table 1). Both of these classes had a higher proportion of patients with comorbidities that did not receive curative treatment, had low education level, and were diagnosed at district hospitals. The median survival time for Class 3 was 2.2 years (IQR: 1.2–4.5 years) and for Class 4 1.6 years (IQR: 0.9–3.9 years) (Table 2). Both classes had a similar proportion that were alive at end of study period, and that died from bladder cancer, but Class 3 had a slightly higher proportion of study participants dying from other causes. Patients in Class 4 treated with radical cystectomy (as compared to no curative treatment) and patients in Class 3 with moderate comorbidity (as compared to no comorbidities), had a lower risk of bladder cancer death (Table S1, for *p* < 0.001). For patients in both classes, several factors were associated to risk of death from bladder cancer and other causes. The HRs for class membership for bladder cancer death were very similar in the two classes: 0.06 and 0.07, which implies that these classes had a hazard rate of about 15 times less for bladder cancer death as those in Class 5. The HR for class membership for other causes of death were 0.31 for Class 3 and 0.16 for Class 4, which gives hazard rates of approximately one third and one sixth respectively as compared to Class 5.

The two smallest classes; Class 1 and Class 2, included totally 7.5% of the studied population and were also more difficult to characterise (Table 1). These classes had a higher proportion woman, comorbidities, lower education level, diagnosed with higher *T* stages and not receiving curative treatment. Class 1 had a median survival time of 0.8 years (IQR 0.2–2.1 years) and Class 2 2.3 years (IQR 1.2–5.2 years). These classes had HRs for class membership for the endpoints of 0.07–0.21, except for HR for Class 1 for death for other causes that were 0.81. This implies that Class 1 have approx. only 1.25 lower hazard rate of death from other causes, that is substantially closer to Class 5, as compared to the estimates from all other classes.

Regional distribution of the classes is shown in Table S2. No significant differences in classes were found between regions.

## 4 DISCUSSION

### 4.1 Key findings

Data from a clinical database merged with other national registers and a latent class proportional hazards model could identify six distinct latent classes of MIBC patients. The largest, and most frail, class corresponded to nearly half of the studied population and was characterised by a somewhat higher proportion of women, older age at diagnosis, more advanced bladder cancer and a higher proportion not receiving curative treatment. Despite this class having the worst prognosis, curative treatment by radical cystectomy or radiotherapy were associated with decreased risk for both death from bladder cancer and other causes. The second largest class corresponded to nearly one fourth of the studied population and was characterised by a higher proportion of men, younger age at diagnosis, higher education level, being diagnosed at a university hospital, less comorbidities, less advanced bladder cancer and a higher proportion treated with radical cystectomy. In both these classes, higher T, N and M stages, and more comorbidities were associated to higher risk of death. In addition to these two major classes, two smaller classes of around 9%–10% each were found; these classes were more difficult to characterise, but both had a higher proportion of patients diagnosed at district hospitals, with low education level, more comorbidities and not receiving curative treatment. Lastly, two even smaller classes of 3%–4% each were found, with a larger proportion woman and patients diagnosed with higher T stages. These two smaller classes had a better bladder cancer and overall survival than the largest class, but the smallest class had almost as high risk of death from other causes as the largest and most frail class. There were no tangible differences in the class‐distribution over health care regions.

### 4.2 Strengths and limitations

We used advanced modelling based on high‐quality register data from an entire nation^8^ to map inter‐patient heterogeneity accounting for competing risks in a systematic approach which avoids violation of underlying statistical assumptions. This new method included an estimation of frailty and detected six latent classes in the study population of patients diagnosed with MIBC with class‐specific risk factors of death from bladder cancer and other causes. We used information on the patients out‐and in‐patient hospital care as a measure of the comorbidity as a covariate; comorbidities diagnosed in primary care or other healthcare institutions were not captured. Also, the original definition of CCI = 0 includes a wide range of health statuses. Thus, the CCI = 0 category may include a group of patients with medical contraindications to major surgery and/or chemotherapy. We had no information on smoking status or body mass index, factors that have been previously associated with molecular subtype of MIBC.^4^ However, identification of molecular subtypes was beyond the scope of this study as we had no access to pathological or molecular details of the bladder tumours,^1^ nor genetic factors or epistasis,^13^ albeit that could have been used to further characterise the heterogeneity found in the study.

### 4.3 Interpretation

The data included in the study are ‘real‐world data’ and reflect characteristics in the entire bladder cancer population in Sweden with a mixture of patients with widely different prognosis and health status—many of them only offered palliative treatment. Thus, this population differs from many other institutional cohorts where a majority were offered treatment with curative intent. The follow up times in the current study are in line with data already published from the BladderBaSe^14^, ^15^ and in similar settings in other countries.^16^

The classes detected in our study had distinctly different bladder cancer prognosis as well as different risks of dying from other causes. These results indicates that there is meaningful information in a routinely collected database that potentially be used further in observational studies or comparisons of performance between counties, regions, or hospitals, as the class characterisation may show differences in ‘case‐mix’ that explain differences in survival. T stage at diagnosis was an influential prognostic marker in the four largest classes and M and N stage in several of the classes, indicating that early detection is important for patients with MIBC.^17^

Moreover, in a future perspective our results could give clues for clinical management or for further risk stratification in interventions or in more specific observational research. For example, the largest class had the worst prognosis, but seemed on the other hand to respond well to treatment with curative intent. Interestingly, still 59% of this class was not offered such treatment. These numbers are in line with other studies reporting that treatment with curative treatment is underused, particular in the elderly and with low socioeconomic status.^18^ A large proportion of patients in this class had missing N or M stage. Likely, a proportion had M1 disease that was not recorded; in patients with bad general health status or high age that preclude any treatment except best supportive care, verification of N and M‐status is often omitted and detailed status therefore not reported. We cannot from this study deduct if abstaining from radical treatment is rationally associated with treatment decisions related to the high frailty or if it also in some cases represents missed opportunities. In a wider perspective, the study is also a test of a method to find clinically relevant subclasses in cancer patients in general.

A further example of research questions that arises from the behaviour of the subclasses is the seemingly contra intuitive results for patients in Class 2. To interpret their outcome, one needs to consider that our model allocates individuals into classes accounting for both known and unknown covariates. Based on their known covariates, we would expect patients in Class 2 to have poorer outcomes. However, they still seem to be robust with respect to a longer survival time, hence, this must be associated with one or more unknown factor(s) not captured with our data‐points. We can only speculate if this may be a factor related (i) to their age at diagnosis, that is the sub population in Class 2 which are older than 75 may be more robust than the group below 75 years, simply because they have not died already, due to unknown factors that could be related to genes or lifestyle or other factors that is associated with longevity, or to (ii) a factor that may be contraindicative to curative treatment but associated with longevity, or to (iii) another molecular, clinical, or biological factor that is associated with a more slowly growing tumour and thus a better prognosis. One may argue that a small sub‐class should be integrated with a larger one. However, that may hinder further investigations of an interesting sub‐class and may dilute the characterisation of the larger class.

We did not intend to study the influence of molecular bladder cancer subtypes. However, our findings lend themselves to generate hypothesis of the association and/or interaction between clinical data of the kind we used and molecular subtype/class. Interestingly, a consensus article recently agreed on six molecular subtypes from transcriptomic data from 1750 MIBC patients.^19^ That study also reported that the largest class (basal/squamous, 35% of the studied population) were more common among women and had a median survival time of 1.2 years. The second largest class were luminal papillary subtype comprising of 24% of all patients. Associations between gender and molecular subtypes have also been reported in later studies,^5^ with higher rates of basal/squamous subtype in women than men, whereas men had higher proportions of the luminal subtypes. Other reports have shown that patients with basal‐like tumours compared to those with luminal subtypes were more likely to be older at diagnosis, obese and have started smoking at a younger age (*n* = 372).^4^ Hence, results in our study are compatible with previous research with respect to number of classes, gender, ages and survival pattern for the largest class.

Larger studies investigating heterogeneity of characteristics of patients with MIBC at diagnosis are sparse, likewise are studies of heterogeneity of progression and responses to treatment. Two studies have reported different responses to neoadjuvant chemotherapy for different molecular subtypes,^20^, ^21^ but to our knowledge, no studies have assessed other treatments or factors related to progression for latent classes of MIBC. Also, to our knowledge, there are no other study of risk factors, treatments, progression and survival that have accounted for frailty in assessment of separate classes of MIBC.

### 4.4 Generalisability

Our study originates from high‐quality register data from an entire nation during more than 15 years follow up, and our main results should be generalisable to regions with similar patterns in risk factors, diagnostic routines, treatment guidelines and prognosis. We found practically no geographical differences in distribution of classes. The patients contributing to this study have mostly north European ancestry and regional differences may be larger in populations where for example the aetiology of the bladder cancer would differ more than in Sweden. Example our results are probably not generalizable to MIBC patients infected by *Schistosoma haematobium* as this infection not is endemic in Sweden.

The novel analysis used in this study is generalisable to cohort studies in a broad spectrum of diseases to analyse inter‐patient heterogeneity in survival. Moreover, it also considers risk of competing events which is important since many patient groups are elderly and frail. Under the hypothesis that class‐specific characteristics for a certain disease differ much between different settings, our study shows that this analysis can be applied to find the relevant classes for the specific situation if baseline data from a clinical register is present.

## 5 CONCLUSION

We identified six distinct latent classes of MIBC patients and their class specific differences and association to risk of death from bladder cancer and other causes. Our results are compatible with data from other investigations of heterogeneity of MIBC and raises research questions to further understand and characterise the subclasses of MIBC patients. The method used can be expanded to include biomarkers and molecular data to probe if precision medicine^22^ can be further used to individually tailor treatment of MIBC. These findings also have implications for risk stratification in both interventional and observational research.

## AUTHOR CONTRIBUTIONS

**Christel Häggström:** Resources (equal); visualization (equal); writing – original draft (equal); writing – review and editing (equal). **Mark Rowley:** Resources (equal); software (equal); validation (equal); visualization (equal); writing – review and editing (equal). **Fredrik Liedberg:** Conceptualization (equal); resources (equal); supervision (equal); writing – review and editing (equal). **Anthony Coolen:** Software (equal); supervision (equal); validation (equal); visualization (equal); writing – review and editing (equal). **Lars Holmberg:** Resources (equal); software (equal); supervision (equal); validation (equal); writing – original draft (equal); writing – review and editing (equal).

## FUNDING INFORMATION

This work was supported by the Swedish Cancer Society (CAN 2013/472 and CAN 2017/278 to LH).

## CONFLICT OF INTEREST STATEMENT

The authors declare no conflict of interest.

## Supporting information


**Table S1.** **Table S2**.

## Data Availability

The data that support the findings of this study may be requested from the included Swedish registers. Ethical and legal restrictions may apply to the availability of these data, and study‐specific ethical permissions are required.

## References

[1] Zhang Y , Zhu C , Curado MP , Zheng T , Boyle P . Changing patterns of bladder cancer in the USA: evidence of heterogeneous disease. BJU Int. 2012;109(1):52‐56. 10.1111/j.1464-410X.2011.10283.x PMC3218244 21592300

[2] Viswambaram P , Hayne D . Gender discrepancies in bladder cancer: potential explanations. Expert Rev Anticancer Ther. 2020;20(10):841‐849. 32896196 10.1080/14737140.2020.1813029

[3] Andreassen BK , Myklebust TA , Haug ES . Crude mortality and loss of life expectancy of patients diagnosed with urothelial carcinoma of the urinary bladder in Norway. Scand J Urol. 2017;51(1):38‐43. 28084860 10.1080/21681805.2016.1271354

[4] Sun X , Hoadley KA , Kim WY , Furberg H , Olshan AF , Troester MA . Age at diagnosis, obesity, smoking, and molecular subtypes in muscle‐invasive bladder cancer. Cancer Causes Control. 2017;28(6):539‐544. 28321693 10.1007/s10552-017-0885-z PMC5477976

[5] de Jong JJ , Boormans JL , van Rhijn BWG , et al. Distribution of molecular subtypes in muscle‐invasive bladder cancer is driven by sex‐specific differences. Eur Urol Oncol. 2020;3(4):420‐423. 32205136 10.1016/j.euo.2020.02.010

[6] Burg ML , Clifford TG , Bazargani ST , et al. Frailty as a predictor of complications after radical cystectomy: a prospective study of various preoperative assessments. Urol Oncol. 2019;37(1):40‐47. 30448327 10.1016/j.urolonc.2018.10.002

[7] Chappidi MR , Kates M , Patel HD , et al. Frailty as a marker of adverse outcomes in patients with bladder cancer undergoing radical cystectomy. Urol Oncol. 2016;34(6):256.e1‐6. 10.1016/j.urolonc.2015.12.010 PMC4875870 26899289

[8] Haggstrom C , Liedberg F , Hagberg O , et al. Cohort profile: the Swedish national register of urinary bladder cancer (SNRUBC) and the Bladder Cancer Data Base Sweden (BladderBaSe). BMJ Open. 2017;7(9):e016606. 10.1136/bmjopen-2017-016606 PMC5623498 28963292

[9] Charlson ME , Pompei P , Ales KL , Mackenzie CR . A new method of classifying prognostic Co‐morbidity in longitudinal‐studies–development and validation. J Chronic Dis. 1987;40(5):373‐383. 3558716 10.1016/0021-9681(87)90171-8

[10] Berglund A , Garmo H , Tishelman C , Holmberg L , Stattin P , Lambe M . Comorbidity, treatment and mortality: a population based cohort study of prostate cancer in PCBaSe Sweden. J Urol. 2011;185(3):833‐839. 21239002 10.1016/j.juro.2010.10.061

[11] Sweden S . Longitudinal integration database for health insurance and labor market studies. 2010 Accessed October 18 2022. https://www.scb.se/en/services/ordering‐data‐and‐statistics/ordering‐microdata/vilka‐mikrodata‐finns/longitudinella‐register/longitudinal‐integrated‐database‐for‐health‐insurance‐and‐labour‐market‐studies‐lisa/

[12] Rowley M , Garmo H , Van Hemelrijck M , et al. A latent class model for competing risks. Stat Med. 2017;36(13):2100‐2119. 28233395 10.1002/sim.7246

[13] Urbanowicz RJ , Andrew AS , Karagas MR , Moore JH . Role of genetic heterogeneity and epistasis in bladder cancer susceptibility and outcome: a learning classifier system approach. J Am Med Inform Assoc. 2013;20(4):603‐612. 23444013 10.1136/amiajnl-2012-001574 PMC3721175

[14] Häggström C , Garmo H , de Luna X , et al. Survival after radiotherapy versus radical cystectomy for primary muscle‐invasive bladder cancer: a Swedish nationwide population‐based cohort study. Cancer Med. 2019;8(5):2196‐2204. 30938068 10.1002/cam4.2126 PMC6536982

[15] Westergren D‐O , Gårdmark T , Lindhagen L , Chau A , Malmström P‐U . A nationwide, population based analysis of patients with organ confined, muscle invasive bladder cancer not receiving curative intent therapy in Sweden from 1997 to 2014. J Urol. 2019;202(5):905‐912. 31144594 10.1097/JU.0000000000000350

[16] Møller CT , Fosså SD , Tafjord G , Babigumira R , Berge V , Andreassen BK . Primary versus secondary muscle‐invasive bladder cancer: survival after curative treatment. Scand J Urol. 2022;56(3):214‐220. 35506475 10.1080/21681805.2022.2056633

[17] Russell B , Liedberg F , Khan MS , et al. A systematic review and meta‐analysis of delay in radical cystectomy and the effect on survival in bladder cancer patients. Eur Urol Oncol. 2020;3(2):239‐249. 31668714 10.1016/j.euo.2019.09.008

[18] Gray PJ , Fedewa SA , Shipley WU , et al. Use of potentially curative therapies for muscle‐invasive bladder cancer in the United States: results from the national cancer data base. Eur Urol. 2013;63(5):823‐829. 23200811 10.1016/j.eururo.2012.11.015

[19] Kamoun A , de Reynies A , Allory Y , et al. A consensus molecular classification of muscle‐invasive bladder cancer. Eur Urol. 2020;77(4):420‐433. 31563503 10.1016/j.eururo.2019.09.006 PMC7690647

[20] Seiler R , Ashab HAD , Erho N , et al. Impact of molecular subtypes in muscle‐invasive bladder cancer on predicting response and survival after neoadjuvant chemotherapy. Eur Urol. 2017;72(4):544‐554. 28390739 10.1016/j.eururo.2017.03.030

[21] Sjodahl G , Abrahamsson J , Holmsten K , et al. Different responses to neoadjuvant chemotherapy in urothelial carcinoma molecular subtypes. Eur Urol. 2022;81(5):523‐532. 34782206 10.1016/j.eururo.2021.10.035

[22] National Research Council . Toward precision medicine: building a knowledge network for biomedical research and a new taxonomy of disease. National Academies Press; 2011. 22536618

