# Supplementary material for: Latent heterogeneity of muscle‐invasive bladder cancer in patient characteristics and survival: A population‐based nation‐wide study in the Bladder Cancer Data Base Sweden (BladderBaSe)
Source: Cancer Med. 2023 Apr 25;12(12):13856–64. doi: 10.1002/cam4.5981 (PMC10315802; doi:10.1002/cam4.5981)
Supplement: Supplementary file 1 — Table S1. Table S2. [file CAM4-12-13856-s001.pdf]

**Supplement Table 1.** Class-specific hazard ratios for covariates included in the analysis, separately for each endpoint.

| **Endpoint 1: Bladder cancer death** | | | | | | |
|---|---|---|---|---|---|---|
| | **Class 1**<br><br>**(N=341, 3.5%)** | **Class 2**<br><br>**(N=384, 4.0%)** | **Class 3**<br><br>**(N=954, 9.9%)** | **Class 4**<br><br>**(N=920, 9.5%)** | **Class 5**<br><br>**(N=4795, 49.7%)** | **Class 6**<br><br>**(N=2259, 23.4%)** |
| | HR (95% CI) | HR (95% CI) | HR (95% CI) | HR (95% CI) | HR (95% CI) | HR (95% CI) |
| Gender | | | | | | |
| Women | 1.00 | 1.00 | 1.00 | 1.00 | 1.00 | 1.00 |
| Men | NS | 2.16 (1.23,3.79) | NS | 0.60 (0.37,0.99) | 0.85 (0.74,0.98) | 0.50 (0.29,0.86) |
| Treatment | | | | | | |
| No curative treatment | 1.00 | 1.00 | 1.00 | 1.00 | 1.00 | 1.00 |
| Radical cystectomy | NS | NS | NS | 0.04 (0.01,0.12) | 0.21 (0.16,0.26) | 0.30 (0.10,0.87) |
| Radiotherapy | NS | NS | 0.47 (0.26,0.85) | NS | 0.47 (0.40,0.57) | NS |
| Age (continous) | | | | | | |
| Per year increment | 4.99 (1.14,21.80) | NS | 3.14 (1.11,8.83) | 2.62 (1.48,4.64) | 1.23 (1.02,1.47) | NS |
| Calendar time | | | | | | |
| yrs 2010-2014 | 1.00 | 1.00 | 1.00 | 1.00 | 1.00 | 1.00 |
| yrs 1997-2000 | NS | NS | NS | NS | 0.71 (0.57,0.87) | NS |
| yrs 2001-2005 | 0.21 (0.07,0.60) | NS | NS | NS | NS | NS |
| yrs 2006-2009 | NS | NS | NS | NS | NS | 2.27 (1.35,3.81) |
| Education level | | | | | | |
| Low | 1.00 | 1.00 | 1.00 | 1.00 | 1.00 | 1.00 |
| High | NS | NS | NS | NS | NS | 0.63 (0.44,0.92) |
| Intermediate | NS | NS | NS | 2.83 (1.62,4.96) | NS | 0.42 (0.22,0.80) |
| CCI | | | | | | |
| CCI=0 | 1.00 | 1.00 | 1.00 | 1.00 | 1.00 | 1.00 |
| CCI=1 | NS | NS | 2.05 (1.01,4.15) | NS | NS | 2.24 (1.09,4.61) |
| CCI=2 | NS | NS | 0.20 (0.09,0.45) | NS | NS | NS |

| | | | | | | |
|---|---|---|---|---|---|---|
| CCI=3+ | NS | 0.50 (0.28,0.90) | NS | 1.92 (1.07,3.45) | NS | 4.09 (2.73,6.13) |
| T stage | | | | | | |
| T2 | 1.00 | 1.00 | 1.00 | 1.00 | 1.00 | 1.00 |
| T3 | NS | NS | 4.91 (2.57,9.39) | NS | 1.69 (1.44,1.97) | 2.97 (1.61,5.48) |
| T4 | NS | NS | 3.94 (1.93,8.04) | 3.91 (2.61,5.88) | 1.58 (1.28,1.94) | 4.38 (2.50,7.67) |
| Grade | | | | | | |
| G2 | 1.00 | 1.00 | 1.00 | 1.00 | 1.00 | 1.00 |
| G3 | NS | NS | NS | 5.58 (2.42,12.89) | 1.19 (1.03,1.38) | NS |
| GX/missing | 2.62 (1.38,4.96) | NS | NS | NS | NS | NS |
| Hospital size | | | | | | |
| District hospital | 1.00 | | 1.00 | 1.00 | 1.00 | 1.00 |
| Region hospital | 3.85 (1.99,7.46) | NS | NS | NS | 0.76 (0.61,0.95) | NS |
| University hospital | NS | NS | NS | NS | NS | NS |
| M stage | | | | | | |
| M0 | 1.00 | 1.00 | 1.00 | 1.00 | 1.00 | 1.00 |
| M1 | NS | 6.47 (3.49,12.02) | 3.57 (2.18,5.85) | NS | 1.49 (1.22,1.81) | NS |
| MX | NS | NS | NS | NS | NS | NS |
| N stage | | | | | | |
| N0 | 1.00 | 1.00 | 1.00 | 1.00 | 1.00 | 1.00 |
| NX | 4.13 (1.84,9.27) | NS | 3.43 (1.58,7.47) | NS | NS | NS |
| N + | NS | 2.40 (1.44,4.00) | 2.78 (1.46,5.29) | NS | 1.32 (1.14,1.53) | 2.06 (1.23,3.46) |

| Endpoint 2: Other causes of death | | | | | | |
|---|---|---|---|---|---|---|
| | Class 1<br><br>(N=341, 3.5%) | Class 2<br><br>(N=384, 4.0%) | Class 3<br><br>(N=954, 9.9%) | Class 4<br><br>(N=920, 9.5%) | Class 5<br><br>(N=4795, 49.7%) | Class 6<br><br>(N=2259, 23.4%) |
| | HR (95% CI) | HR (95% CI) | HR (95% CI) | HR (95% CI) | HR (95% CI) | HR (95% CI) |
| Gender | | | | | | |
| Women | 1.00 | 1.00 | 1.00 | 1.00 | 1.00 | 1.00 |
| Men | 0.45 (0.25,0.83) | NS | NS | NS | NS | NS |
| Treatment | | | | | | |
| No curative treatment | 1.00 | 1.00 | 1.00 | 1.00 | 1.00 | 1.00 |
| Radical cystectomy | NS | NS | NS | NS | 0.19 (0.07,0.48) | NS |
| Radiotherapy | NS | NS | NS | NS | 0.28 (0.14,0.57) | NS |
| Age (continous) | | | | | | |
| Per year increment | 0.35 (0.19,0.65) | 10.64 (2.71,41.83) | 6.67 (3.47,12.84) | 7.39 (2.51,21.74) | NS | 39.93 (15.79,100.98) |
| Calendar time | | | | | | |
| yrs 2010-2014 | 1.00 | 1.00 | 1.00 | 1.00 | 1.00 | 1.00 |
| yrs 1997-2000 | NS | 6.11 (2.25,16.55) | NS | 6.36 (2.20,18.37) | NS | NS |
| yrs 2001-2005 | NS | NS | NS | 7.64 (1.92,30.45) | NS | NS |
| yrs 2006-2009 | NS | 9.20 (3.80,22.29) | NS | 4.15 (1.51,11.37) | NS | 0.24 (0.09,0.66) |
| Education level | | | | | | |
| Low | 1.00 | 1.00 | 1.00 | 1.00 | 1.00 | 1.00 |
| High | NS | 3.26 (1.57,6.80) | 0.52 (0.28,0.97) | NS | NS | NS |
| Intermediate | 0.43 (0.21,0.92) | NS | NS | NS | NS | NS |
| CCI | | | | | | |
| CCI=0 | 1.00 | 1.00 | 1.00 | 1.00 | 1.00 | 1.00 |

| | | | | | | |
|---|---|---|---|---|---|---|
| CCI=1 | 2.32 (1.02,5.25) | NS | NS | NS | 1.49 (1.03,2.14) | NS |
| CCI=2 | NS | NS | 2.80 (1.71,4.60) | 8.10 (4.99,13.14) | NS | 0.46 (0.22,0.95) |
| CCI=3+ | NS | 4.85 (2.14,10.97) | 2.98 (1.95,4.54) | 2.51 (1.12,5.62) | 2.43 (1.42,4.16) | 0.43 (0.20,0.90) |
| T stage | | | | | | |
| T2 | 1.00 | 1.00 | 1.00 | 1.00 | 1.00 | 1.00 |
| T3 | 2.59 (1.56,4.30) | NS | NS | NS | NS | NS |
| T4 | 2.36 (1.10,5.09) | NS | NS | NS | NS | NS |
| Grade | | | | | | |
| G2 | 1.00 | 1.00 | 1.00 | 1.00 | 1.00 | 1.00 |
| G3 | NS | 0.46 (0.30,0.70) | NS | NS | NS | NS |
| NA | NS | NS | NS | NS | NS | NS |
| Hospital size | | | | | | |
| District hospital | 1.00 | 1.00 | 1.00 | 1.00 | 1.00 | 1.00 |
| Region hospital | 2.34 (1.12,4.90) | 2.29 (1.18,4.45) | NS | NS | NS | NS |
| University hospital | NS | NS | NS | 0.25 (0.06,0.93) | NS | NS |
| M stage | | | | | | |
| M0 | 1.00 | 1.00 | 1.00 | 1.00 | 1.00 | 1.00 |
| M1 | NS | NS | NS | NS | NS | 4.42 (2.16,9.07) |
| MX | NS | NS | NS | NS | NS | NS |
| N stage | | | | | | |
| N0 | 1.00 | 1.00 | 1.00 | 1.00 | 1.00 | 1.00 |
| NX | NS | 0.45 (0.23,0.85) | NS | 2.71 (1.49,4.94) | 3.43 (1.84,6.40) | NS |
| N + | NS | NS | 4.71 (2.93,7.59) | 0.31 (0.10,0.95) | NS | NS |

Hazard ratios with p-value of less than 0.05 are included in the tables, and hazard ratios with p-values less than 0.001 are highlighted in red (increased hazard) and green (reduced hazard). NS= not significant.

**Supplement Table 2.** Regional distribution of the six latent classes from the competing risk latent class analysis.

| | Health care region, (n, %) | | | | | |
|---|---|---|---|---|---|---|
| | **Sthlm (N=1514, 16%)** | **South (N=2118, 22%)** | **Southeast (N=1128, 12%)** | **Mid (N=2298, 24%)** | **West (N=1588, 16%)** | **North (N=1007, 10%)** |
| **Class 1** | 42 (3) | 71 (3) | 45 (4) | 88 (4) | 49 (3) | 46 (5) |
| **Class 2** | 67 (4) | 79 (4) | 44 (4) | 96 (4) | 61 (4) | 37 (4) |
| **Class 3** | 144 (10) | 221 (10) | 113 (10) | 214 (9) | 157 (10) | 105 (10) |
| **Class 4** | 154 (10) | 197 (9) | 102 (9) | 243 (11) | 137 (9) | 87 (9) |
| **Class 5** | 731 (48) | 1043 (49) | 565 (50) | 1125 (49) | 831 (52) | 500 (50) |
| **Class 6** | 376 (25) | 507 (24) | 259 (23) | 532 (23) | 353 (22) | 232 (23) |
| **Survival time (years)*** Median (IQR) | 0.8 (0.4-1.9) | 0.8 (0.3-1.9) | 0.8 (0.4-1.8) | 0.8 (0.4-1.9) | 0.7 (0.3-1.6) | 0.8 (0.3-1.8) |

*Excluding study participants alive at end of study
